# Can pre-operative intraarticular injection predict pain relief after total hip arthroplasty?

**DOI:** 10.1186/s12891-022-05969-4

**Published:** 2023-01-09

**Authors:** Thorsten Jentzsch, Yann K. Meyer, Ines Unterfrauner, Andrea B. Rosskopf, Christian W. Pfirrmann, Patrick O. Zingg

**Affiliations:** 1 Department of Orthopaedics, Balgrist University Hospital, University of Zurich, Zurich, Switzerland; 2 Faculty of Medicine, University of Zurich, Zurich, Switzerland; 3 Department of Radiology, Balgrist University Hospital, Zurich, Switzerland

**Keywords:** Pain, Injection, Steroids, Anesthetics, Local, Hip, Arthroplasty

## Abstract

**Background:**

To study if pain relief after injection and arthroplasty correlate.

**Methods:**

A retrospective cohort study included consecutive patients (*n* = 88; median age 64 (interquartile range (IQR) 22) years, 49 (56%) females) that received fluoroscopic-guided intra-articular hip injection with contrast agent, anaesthetic (diagnostic), and corticosteroid (therapeutic) before implantation of primary total hip arthroplasty. Pain scores were assessed pre-injection, post-injection after 15 min (diagnostic phase) at first clinical follow up (therapeutic phase; median 2 (IQR 2) months), and postoperatively (last follow up (median 15 (IQR 5) months)). Responders had reduction in pain score ≥ 20 (numeric rating scale 0–100) points. The primary outcome was the same (or inverse) response to injection and arthroplasty.

**Results:**

The median pain scores were higher pre-injection (68 (IQR 30) points) compared to the diagnostic phase (18 (IQR 40) points; *p* < 0.001), therapeutic phase (50 (IQR 40) points; *p* < 0.001), and post-operatively (2 (IQR 15) points; *p* < 0.001). On the one hand, 69 (78%) cases had the same response in the diagnostic phase and post-operatively (rho = 0.58; *p* < 0.001; sensitivity 83%); on the other hand 32 (36%) cases had the same response in the therapeutic phase and post-operatively (rho = 0.25; *p* < 0.001; sensitivity 33%). Furthermore, 57% and 91% of patients had an even better response post-operatively than in the diagnostic and therapeutic phases.

**Conclusions:**

Pre-operative intraarticular injection can predict pain relief after primary total hip arthroplasty. A positive response to hip arthroplasty may be better predicted by the response to local anaesthetic (diagnostic phase) than corticosteroids. Most patients (91%) with osteoarthritis may expect better pain relief after arthroplasty compared to the therapeutic phase after injection.

## Introduction

Intra-articular steroid injection for osteoarthritis of the hip is recommended by several international guidelines (e.g. European League Against Rheumatism) [1, 2]. It is usually suggested in non-responders to oral pain medication and commonly performed using imaging guidance. Injections often combine local anesthetics to confirm the joint as source of pain (immediate diagnostic phase) and steroids to treat a painful joint (mid- to long-term therapeutic phase). The expected results can be independent from the radiographic severity of osteoarthritis [3]. The risk of complications is rare (e.g. infection in < 0.001%) [4]. A recent systematic review by McCabe et al. reported efficacious short-term pain relief after injection of intraarticular steroids for osteoarthritis [5]. One of the conclusions was that, due to the poor evidence, further studies were called for to verify the effects of injections.

If patients still continue to experience pain after injection(s) due to their hip osteoarthritis, they may opt to undergo total hip arthroplasty. The long-term outcome of total hip arthroplasty is very good and the satisfaction rates (92%) are usually superior to other common orthopaedic surgeries (e.g. transforaminal lumbar interbody fusion (86%) and hallux valgus correction (77%)) [6]. Although known at other regions (e.g. the cervical spine), it would also be interesting to know if the amount of pain reduction after injection and total hip arthroplasty are correlated [7]. This is of clinical importance, not only to be able to discriminate different etiologies of hip pain (such as abductor insufficiency or lumbar radiculopathy) in discrepant findings between the clinical findings and morphological radiographic changes, but also to manage the expectations of patients and orthopaedic surgeons for the outcome after hip arthroplasty. At our institution, we are careful to proceed with hip arthroplasty if a patient did not benefit from at least the local anaesthetic with a substantial pain reduction because surgeons may be worried that the hip joint may not be the main driver of hip pain if the clinical presentation and radiographs are ambiguous. On the other hand, if a patient benefits from an injection in a case with ambiguous clinical presentation and radiographic changes, we feel more comfortable in proceeding with hip arthroplasty.

This study hypothesized that pain relief after injection and arthroplasty correlate with each other. Furthermore, it was opted to find out if the response to hip arthroplasty can be better predicted by the diagnostic or therapeutic response to an injection.

## Materials and methods

A retrospective cohort study was conducted at a single institution between 2016–2018. The local ethics committee provided a waiver for this study (BASEC Nr. Req-2018–00,709). This study was performed in accordance with relevant guidelines and regulations (Declarations of Helsinki).

This study included all consecutive patients (*n* = 88) that received fluoroscopic-guided intra-articular injection of the hip with a contrast agent, an anaesthetic (diagnostic), and corticosteroid (therapeutic) in 2016 before subsequent implantation of a primary total hip arthroplasty. In other words, all patients had an injection and hip arthroplasty, independent of the effect of the injection. Patients with a previous hip surgery (*n* = 10) were excluded. The median difference between injection and arthroplasty was 4 (IQR 6) months. The median follow-up was 15 (IQR 5) months.

Pain scores were assessed at baseline (pre-injection), after 15 min (min; = diagnostic phase), at first clinical follow up (after median of 2 (interquartile range (IQR) 2) months; = therapeutic phase) post-injection, and at last follow up (after median of 15 (IQR 5) months) post-operatively. The scores were self-reported and obtained through chart review.

The primary outcome parameter was the similarity of the response to diagnostic and therapeutic injection as well as arthroplasty (i.e. same direction of response versus (vs) different direction). Responders were defined as patients with absolute reduction in pain score ≥ 20 (on a numeric rating scale of 0–100) points, whereby the initially reported NRS from the radiology department (0–10) was multiplied by 10 [8].

The diagnosis of osteoarthritis of the hip was based on self-reported pain at the hip and radiological evidence of osteoarthritis. Pain was graded on a numeric rating scale from 0–100 (worst) [9]. Osteoarthritis was also graded by two independent and trained investigators on antero-posterior radiographs of the hip according to Kellgren and Lawrence from 0–4 (worst), where 0 = normal (no osteoarthritis), 1 = doubtful (uncertain osteophytes), 2 = mild (osteophytes), 3 = moderate (additional moderate joint space narrowing), and 4 = severe (severe joint space narrowing) [10].

**Fig. 1:**
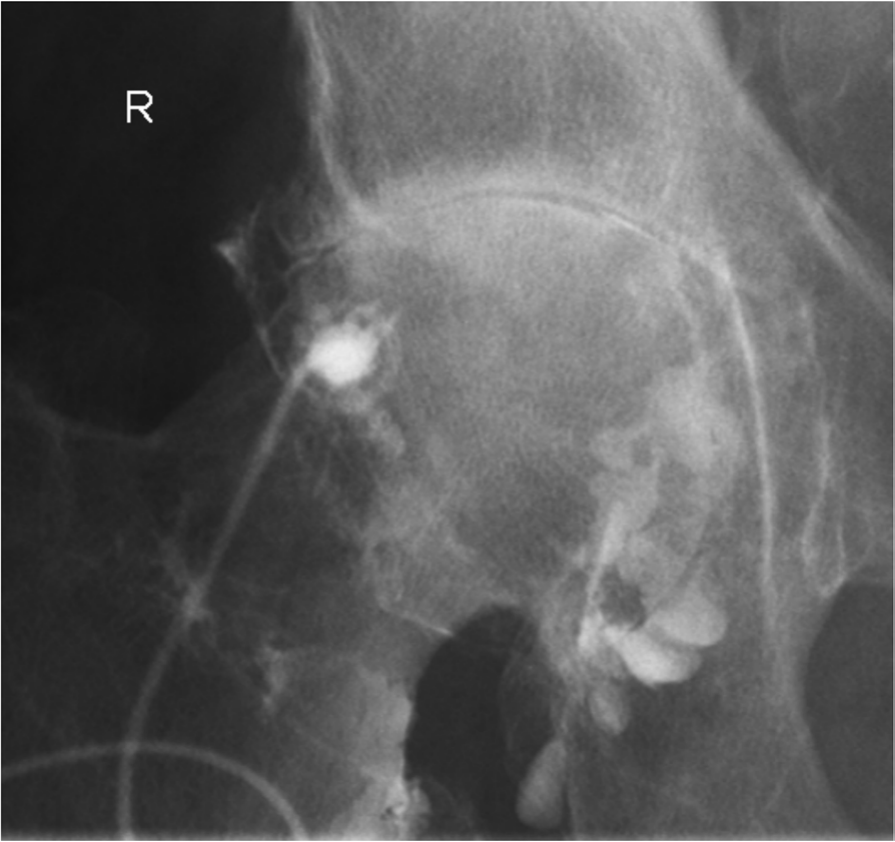
Radiographs of injection and hip arthroplasty. The anteroposterior x-ray shows how contrast agent is injected into the hip joint through a direct anterior approach to the right hip

**Fig. 2:**
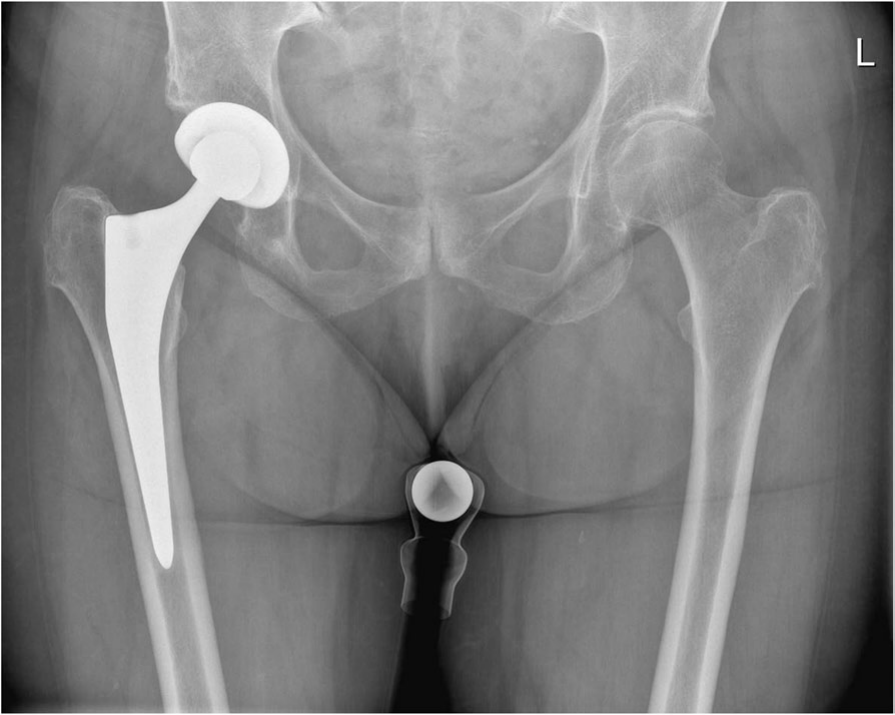
Radiographs of injection and hip arthroplasty. The anteroposterior x-rays shows an implanted primary total hip arthroplasty (Medacta) of the right hip around one year postoperatively

The injection was performed in a standardized fashion similar to the protocol described before [7] after obtaining informed consent. It was done under sterile conditions using chlorhexidine through an anterior approach under x-ray guidance in a fluoroscopy suite by a trained radiologist (Fig. 1). The injection consisted of a prior arthrography with Iopamidol 200 mg/ml (2 ml (ml) Iopamiro 200) and injection of lidocaine 20 mg/ml (5 ml Rapidocain 2%) and triamcinolone 40 mg (mg) (1 ml Triamcort) using a 22-gauge spinal needle. The primary total hip arthroplasties at our institution are commonly performed through a direct anterior approach (Fig. 2).

Data are provided as absolute numbers (percentages) and medians (IQR). The Wilcoxon matched-pairs signed-ranks test, McNemar’s test, and Spearman correlation were used using Stata/IC (version 13.1; StataCorp LP, College Station, TX, USA). Assuming an alpha of 0.05 and a difference in means on the NRS of 20 (standard deviation 20) points between responders and non-responders, 19 patients per group were needed according to a previous calculation by Atchia et al. [11].

## Results

### Participants

The median age was 64 (IQR 22) years and there were 49 (56%) females (*n* = 88). The median Kellgren and Lawrence osteoarthritis grade was 3 (IQR 1).

**Fig. 3:**
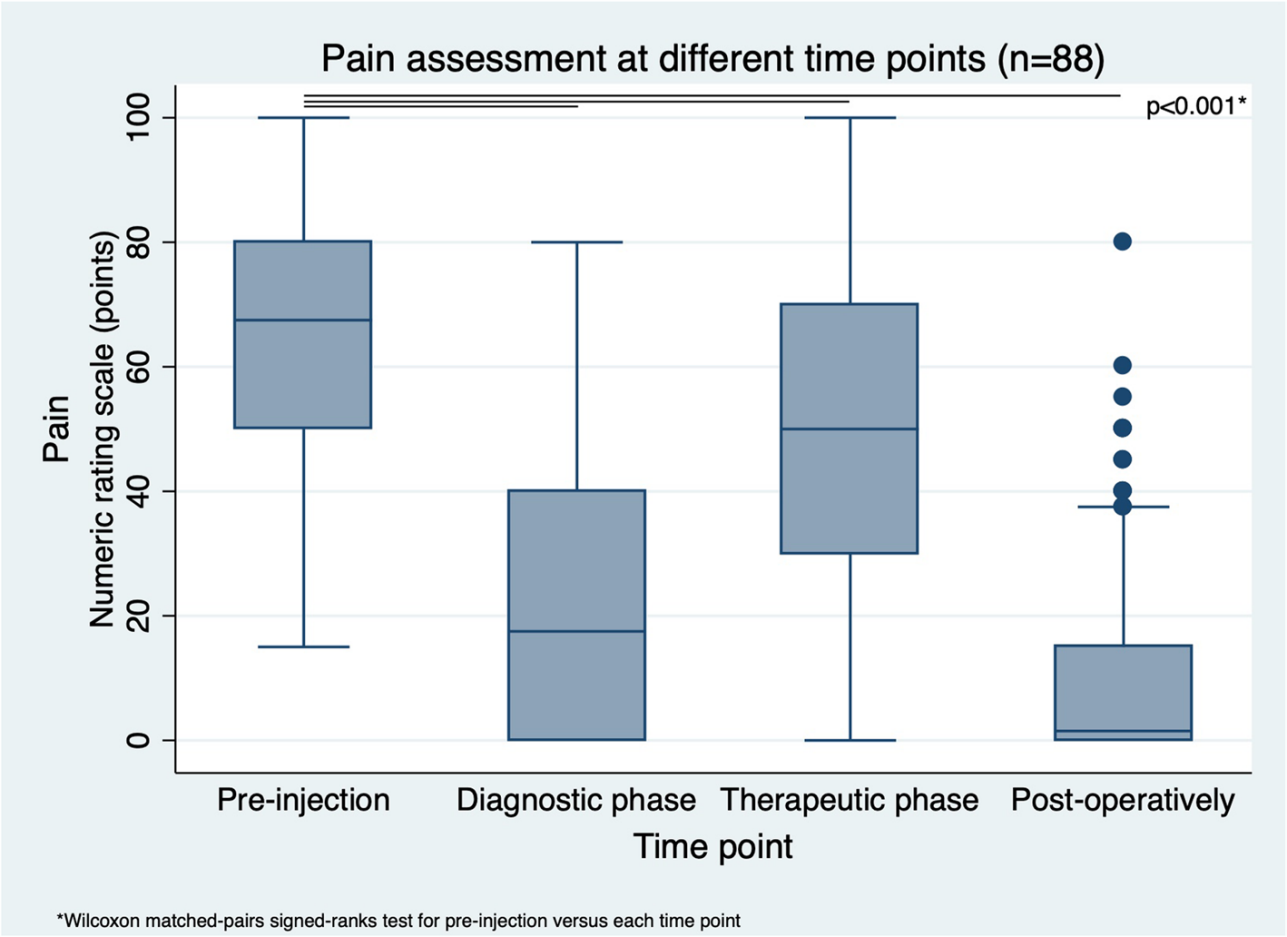
Pain at different time points (*n* = 88). This boxplot shows the pain assessment using a numeric rating scale at different time points (baseline (pre-injection), after 15 min (diagnostic phase) and at first clinical follow (after around 2 months) (therapeutic phase) (post-injection), and at last follow up (after around months) (post-operatively after primary total hip arthroplasty)

The median pain scores were higher at baseline (68 (IQR 30) points) compared to the diagnostic phase (18 (IQR 40) points; *p* < 0.001), in the therapeutic phase (50 (IQR 40) points; *p* < 0.001), and post-operatively after a median of 15 (IQR 5) months (2 (IQR 15) points; *p* < 0.001) (Fig. 3). The pain scores were significantly better for hip arthroplasty than during the diagnostic or therapeutic phase (*p* < 0.001).

Sixty-nine (78%) cases had the same response post-injection in the diagnostic phase and post-operatively (rho = 0.58), compared to 32 (36%) cases with the same response in the therapeutic phase and post-operatively (rho = 0.25) (*p* < 0.001). Furthermore, 57% and 91% of patients had a better response to hip arthroplasty than the diagnostic and therapeutic phases, respectively. For the diagnostic phase, the sensitivity was 83%. For the therapeutic phase, the sensitivity was 33%.

A response (i.e. absolute pain reduction ≥ 20 points) was found post-operatively, but non-response was seen in the diagnostic phase in 14 (16%) patients and in the therapeutic phase in 54 (61%) patients. Contrarily, a non-response was found post-operatively, but a response was observed in the diagnostic phase in five (6%) patients (*p* = 0.458) and in the therapeutic phase in two (2%) patients (*p* = 0.797).

In patients with response to injection, but non-response to hip arthroplasty, the remaining pain was mostly due to muscular dysbalance and potentially due to short-term follow up. The difference between pre- and postoperative pain in these patients ranged within 10 points.

## Discussion

This is the first larger study to show that pre-operative injections can predict pain relief after primary total hip arthroplasty. It was observed that the response during the diagnostic phase, in which the local anaesthetic is effective, is a better predictor of the response to the arthroplasty than the response during the therapeutic phase, in which the corticosteroid is effective. According to the sensitivity, the diagnostic phase can identify 83% of patients who also obtain good pain relief from hip arthroplasty. It was also noted that non-response to injection does not predict non-response to arthroplasty. Overall, injections were not able to reproduce the very good pain relief from arthroplasty.

Up to this study, it had been mostly unknown if the pain relief from injections correlates with the one from hip arthroplasty. To the best of our knowledge, there is only one older study with 42 patients by Crawford et al., which reported that 78% of patients had pain relief from their injection of diagnostic local anaesthetic and 96% had subsequent successful total hip arthroplasty after a minimum of six weeks (but without providing the average follow-up time [12]. Of the 22% of patients that did not have pain relief, 1 patient had unsuccessful total hip arthroplasty, while the remaining 8 patients had other conditions or no organic basis for the pain. While these findings are similar to our study, our findings add important information to the literature in providing a larger sample size, a longer follow-up time, and the effect of therapeutic corticosteroids. Another previous study investigated the correlation between injections and surgery in the spine. Antoniadis et al. found an association (rho = 0.37, *p* = 0.03) between pain relief in the diagnostic phase after cervical nerve root blocks and around two years after decompression in 33 patients [7].

If conservative measures using analgesics and nonsteroidal anti-inflammatory drugs fail for osteoarthritis of the hip, injections are a valuable option as a next step [3]. They usually contain a local anaesthetic and corticosteroids. The local anaesthetic provides immediate pain relief. Clinically, this is also used for diagnostic purposes in complex cases where the clinical symptoms of hip pain are discrepant with morphological radiographic findings in order to discreminate the intraarticular osteoarthritic contribution to the pain from other sources (e.g. abductor insufficiency, trochanteric bursitis or painful radiculopathy). The corticosteroid usually provides longer lasting effects for several months. In a previous clinical trial by Pham et al., the number needed to treat to achieve one responder (i.e., among others, absolute change in pain ≥ 20 points) was 2.4 at two months [8]. The reported effect size was 1.5 and 0.5 on a NRS from 0–10 one and eight weeks post-injection, respectively [11]. The benefits are challenged by recent systematic review of the literature by Kreuz et al., who reported that local anesthetics exhibited a chondrotoxic effect [13]. Since this effect was type-, dose-, and time-dependent, the authors recommended the use of low concentrations (e.g. 0.1 and 0.2% ropivacaine). These effects have also been reported for corticosteroids in a clinical trial by McAlindon et al., where increased cartilage volume loss was found two years after intra-articular triamcinolone injection for knee osteoarthritis [14]. In outpatient clinics, patients usually ask orthopaedic surgeons if they should try an injection before undergoing a surgical procedure such as total hip arthroplasty and if this injection can mimic the postoperative success in pain relief. The current study provides valuable answers to these questions as the immediate effect in the diagnostic phase of an injection points into the direction of the surgical effect, but hip arthroplasty was able to provide the best pain relief. We provide data that shows that patients who benefited from injection with local anaesthetic into the hip joint, are likely to experience substantial pain decrease after hip arthroplasty. This may be used by surgeons in complex cases where the clinical and radiographic presentation are challenging. As reported previously by Werner et al., the authors usually opt to wait, if at all fesible, with implantation of hip arthroplasty for at least three months after injection to decrease the risk of periprosthetic joint infection [15]. In our study, the average pain scores were much lower after hip arthroplasty compared to the ones after injection and pre-operatively and 56% as well as 91% had better pain relief from hip arthroplasty than from diagnostic as well as therapeutic injection, respectively. Therefore, in case injections do not provide enough pain relief, but clinical and radiological findings are in line with hip osteoarthritis and all other potential causes are excluded, patients can mostly rest assured that hip arthroplasty is very likely to be effective anyhow.

There are several potential limitations to this study. Theoretically, the corticosteroid effect may have worn off by the time patients were seen in our outpatient clinic after around two months since the time period of pain relief has been reported as, on average, as two months [16]. During our chart review, we paid attention to the history of the patient and if the effects were described as long-lasting, we documented it this way. The median follow-up after arthroplasty was slightly longer than a year, so these results are applicable to the long-term outcome after hip arthroplasty, since Patil et al. have described that patient outcome plateaus after around one year [17]. We did not include a control group of patients without injection or hip arthroplasty due to the research question about the correlation of injection and arhtroplasty as well as the retrospective nature of this database and the potential loss of follow-up of patients who may have received surgical treatment at an outside institution, which would have led to misclassification. Although the main focus of this study was not to assess the success of each individual intervention per se, but to compare the effect of injections and arthroplasty, a lack of a control group may miss placebo effects with subsequent regression to the mean, which may be a topic for future studies.

## Conclusion

Pre-operative intraarticular injection can predict pain relief after primary total hip arthroplasty. A positive response to hip arthroplasty may be better predicted by the response to local anaesthetic (diagnostic phase). Most patients (88% and 91%, respectively) with osteoarthritis may expect even better pain relief after total hip arthroplasty compared to the therapeutic phase after injection.

## Data Availability

All data analysed during this study are included in this article.

## Abbreviations

IQR: Interquartile range
NRS: Numeric rating scale
ml: Milliliters
